# Comprehensive study of anaplastic large cell lymphoma: clinicopathological features from Indonesia

**DOI:** 10.1186/s13104-025-07354-5

**Published:** 2025-07-09

**Authors:** Agnes Stephanie Harahap, Ivana Santoso, Stefanny Charles, Nadia Ayu Mulansari, Maria Francisca Ham

**Affiliations:** 1 Anatomical Pathology Department, Faculty of Medicine, Universitas Indonesia/Dr. Cipto Mangunkusumo National Central General Hospital, Jakarta, 10430 Indonesia; 2 Human Cancer Research Center-Indonesian Medical Education and Research Institute, Faculty of Medicine, Universitas Indonesia, Jakarta, 10430 Indonesia; 3 Hematology and Medical Oncology Division, Internal Medicine Department, Faculty of Medicine, Universitas Indonesia/Dr. Cipto Mangunkusumo National Central General Hospital, Jakarta, 10430 Indonesia

**Keywords:** Anaplastic large cell lymphoma, ALK-positive ALCL, ALK-negative ALCL, Clinicopathologic characteristics, Immunohistochemistry, Prognostic factors

## Abstract

**Objective:**

Anaplastic large cell lymphoma (ALCL) is a rare and aggressive CD30-positive non-Hodgkin lymphoma with histopathological features overlapping Hodgkin and T-cell lymphomas. ALK-positive ALCL shows a better prognosis than ALK-negative cases, which are often associated with advanced disease. This study evaluates the epidemiological profile of ALCL in Indonesian population and its distinct histopathological characteristics and immunohistochemical expression patterns.

**Results:**

Among 93 ALCL cases (2013–2023) enrolled at Dr. Cipto Mangunkusumo Hospital, 13.9% were primary cutaneous ALCL, while 86.1% were systemic ALCL (consisted of 53.7% ALK-positive and 46.3% ALK-negative). ALK-positive patients were older (*p* = 0.009), with earlier-stage disease (*p* = 0.032) and nodal predilection (*p* = 0.032). ALK-negative cases demonstrated a shorter median survival of 23 months compared to ALK-positive cases of 28 months. Poor outcomes were associated with B symptoms and high Eastern Cooperative Oncology Group Performance Status (ECOG-PS) scores. Given that this study was conducted at a single, government-operated tertiary care teaching hospital in Indonesia’s capital, validation through multicenter prospective studies is warranted to further refine diagnostic and therapeutic strategies.

**Supplementary Information:**

The online version contains supplementary material available at 10.1186/s13104-025-07354-5.

## Introduction

Anaplastic large cell lymphoma (ALCL) is a distinct subtype of mature T-cell non-Hodgkin lymphomas (NHLs), characterized by large pleomorphic malignant cells with horseshoe-shaped nuclei and strong CD30 expression. ALCL is classified into four subtypes: systemic anaplastic lymphoma kinase-positive ALCL (ALK-positive ALCL), systemic anaplastic lymphoma kinase-negative ALCL (ALK-negative ALCL), primary cutaneous ALCL (pc-ALCL), and breast implant-associated ALCL (BIA-ALCL) [1]. Systemic ALCL, the third most common subtype of peripheral T-cell lymphoma (PTCL), accounts for 13.8% of cases worldwide, while pc-ALCL and BIA-ALCL are rarer, representing 1.7% of all PTCL cases and 0.006% of noncutaneous lymphomas, respectively [2, 3]. ALK status significantly influences prognosis and clinical outcomes [4–6]. 

In low to middle income countries, such as Indonesia, ALCL diagnosis and management are complicated by limited access to advanced diagnostic tools [7]. Ethnic and geographical variations in ALCL incidence have been reported, with specific patterns noted in Asian populations. For instance, Malays have the highest age-standardized incidence among major ethnic groups in Singapore [8]. Studies focusing on ALCL have made it difficult to establish accurate epidemiological data on this rare form of lymphoma.

This study aims to investigate the epidemiology, clinicopathological profiles, immunohistochemical (IHC), prognostic factors, and disease outcomes among ALK-positive and ALK-negative subtypes in Indonesia, highlighting the unique and diverse histopathological characteristics of this rare lymphoma to improve diagnosis and treatment in similar settings.

## Methods

### Study design and patient selection

This retrospective study enrolled all cases of ALCL diagnosed at the Department of Anatomical Pathology, Dr. Cipto Mangunkusumo Hospital (CMH), from 2013 to 2023. The inclusion criteria consist of both internal and referral cases of ALCL that have been diagnosed through comprehensive pathological examination and IHC analysis (see Supplementary). Internal cases pertain to patients diagnosed and treated within our institution, irrespective of their residency, whereas referral cases are sent from different regional hospitals in Indonesia (Sumatra, Batam, Java, Bali, Borneo, Sulawesi, and East Nusa Tenggara Island) to our institution for a definitive diagnosis of lymphoma. The exclusion criteria include ALCL cases with inaccessible medical records, incomplete IHC profiles, and inconclusive diagnosis.

### Data collection

Clinical data were collected from medical records, including age, gender, tumor subtype, Ann-Arbor stage, location, presence of B symptoms, and treatment details. Age groups were categorized into children (≤ 19 years) and adults (> 19 years) [9]. Tumor location was classified as nodal or extranodal according to clinical diagnosis established through clinical examination and imaging modalities. The subdivision of nodal and extranodal lymphoma into specified lymph node regions or tissues and/or organs is determined by the topographic sites where biopsies were performed. The performance status, encompassing the patient’s capacity for self-care, daily activities, and physical ability, was evaluated by the Eastern Cooperative Oncology Group Performance Status (ECOG-PS) scale, which ranges from 0 to 5 [10]. The prognosis was evaluated with the international prognostic index (IPI), considering factors such as lactate dehydrogenase (LDH) levels, sites of involvement, stage, and ECOG-PS. ALCL diagnoses were confirmed through a comprehensive IHC panel (see Supplement).

### Pathology assessment

Two hematopathologists evaluated all hematoxylin and eosin (H&E) and IHC slides of each case, according to the latest World Health Organization (WHO) guidelines [1]. Tumor patterns (diffuse, nodular, sinusoidal) and morphological subtypes (common, lymphohistiocytic, giant cell, small cell, CHL-like) were evaluated [4]. ALK staining patterns (cytoplasmic or nuclear/cytoplasmic) and CD30 expression (membrane, cytoplasm, Golgi area) were assessed, along with necrosis, apoptosis, and inflammation.

### Statistical analysis

All research data were analyzed by Microsoft Excel 2023 and SPSS ver.26. Bivariate analysis was conducted using the Chi-square test or Fisher’s exact test to evaluate the distinct clinical and histopathological characteristics between ALK-positive and ALK-negative systemic ALCL. The correlation between prognostic features and disease outcome was analyzed by using Fisher’s exact test. A *p*-value < 0.05 is considered statistically significant.

### Ethics

Ethical approval and informed consent waiver were obtained from the Research Ethics Committee, Faculty of Medicine, Universitas Indonesia-CMH (Protocol: KET-1177/UN2.F1/ETIK/PPM.00.02/2024, ND-604/UN2.F1/ETIK/PPM.00.02/2024).

## Results

**Fig. 1:**
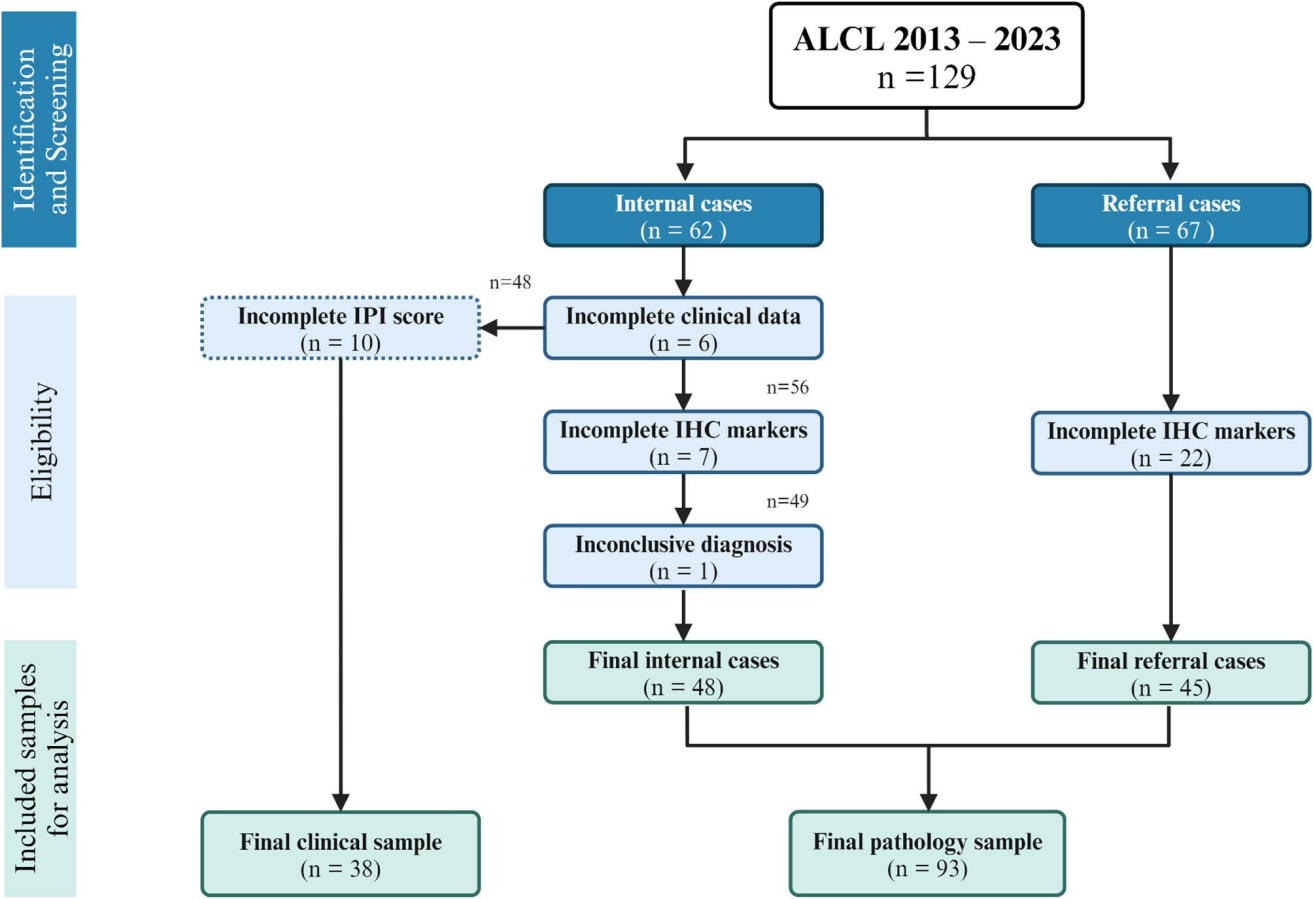
Sample selection process

The study includes 93 ALCL cases, which were selected based on our established inclusion and exclusion criteria, as displayed in Fig. 1. This study analyzed histopathology and immunohistochemistry characteristics in all 93 final pathology cases of ALCL.

### Demographic and clinical profiles

Among 93 cases of ALCL, 13.9% were pc-ALCL and 86.1% were systemic ALCL, which constitutes 53.7% of ALK-positive and 46.3% of ALK-negative. The median age was 41 years (4–74 years), with ALK-positive having a median age of 25 years and ALK-negative was 49 years.

As displayed in Table 1, the neck region was most affected (40%) within nodal involvement, followed by the inguinal, axillary, and submandibular regions, respectively. Additional nodal regions, including the thorax, supraclavicular, and inguinal areas, were identified in a smaller proportion of cases. In contrast, the most common extranodal sites were soft tissues of the upper and lower extremities, accounting for 20% of all cases. Other extranodal sites included the central nervous system, upper respiratory tract, mouth, tonsils, ocular region, stomach, lung, liver, bone, and vertebra-pelvis regions.

Four patients exhibited a familial predisposition to cancer, with a documented history of breast cancer, cervical cancer, brain tumor, or rhabdomyosarcoma in the family. Chemotherapy was given to 14 patients (10 patients had systemic ALCL and 4 patients had pc-ALCL), and radiotherapy was done in 3 patients. The CHOP regimen was frequently given in all cases; the rest of the cases have no recorded treatment details.

**Table 1:** Clinical profiles of cutaneous and systemic anaplastic large cell lymphoma

	ALCL (*N* = 93)				
	Cutaneous ALCL	Systemic ALCL			
	n (%)	ALK-positive n (%)	ALK-negative n (%)	*p-*value	OR (95% CI)
**Age**,** year (n = 93)**					
≤ 19	0 (0.0)	16 (80.0)	4 (20.0)	0.009*	4.9 (1.4–16.3)
> 19	13 (100.0)	27 (45.0)	33 (55.0)		
**Sex (n = 93)**					
Female	6 (46.2)	18 (56.2)	14 (43.8)	0.714*	1.2 (0.5–2.9)
Male	7 (53.8)	25 (52.1)	23 (47.9)		
**Stage (n = 93)**					
Early	1 (7.7)	32 (62.7)	19 (37.3)	0.032*	2.7 (1.1–7.1)
Advanced	12 (92.3)	11 (37.9)	18 (62.1)		
**B symptoms (n = 38)**					
Present	6 (66.7)	10 (47.6)	11 (52.4)	0.682**	1.8 (0.3–9.7)
Absent	3 (33.3)	5 (62.5)	3 (37.5)		
**ECOG– PS (n = 38)**					
0–2	8 (88.9)	12 (57.1)	9 (42.9)	0.427**	2.2 (0.4–11.8)
3–4	1 (11.1)	3 (37.5)	5 (62.5)		
**IPI (n = 38)**					
Low to low-intermediate	4 (44.4)	11 (57.9)	8 (42.1)	0.450**	2.1 (0.4–9.8)
Intermediate-high to high	5 (55.6)	4 (40.0)	6 (60.0)		
**LDH (n = 38)**					
Normal	4 (44.4)	12 (60.0)	8 (40.0)	0.245**	3.0 (0.6–15.6)
High	5 (55.6)	3 (33.3)	6 (66.7)		
**Location (n = 93)**					
Nodal	1 (7.7)	32 (62.7)	19 (37.3)	0.032*	2.8 (1.1–7.1)
Extranodal	12 (92.3)	11 (37.9)	18 (62.1)		

Abbreviations: ALCL, Anaplastic Large Cell Lymphoma; OR, Odd Ratio; 95% CI, 95% Confidence Interval; IPI, International Prognostic Index; LDH, Lactate Dehydrogenase; ECOG-PS, Eastern Cooperative Oncology group Performance Status

*Chi-Square; ** Fisher Exact

**Fig. 2:**
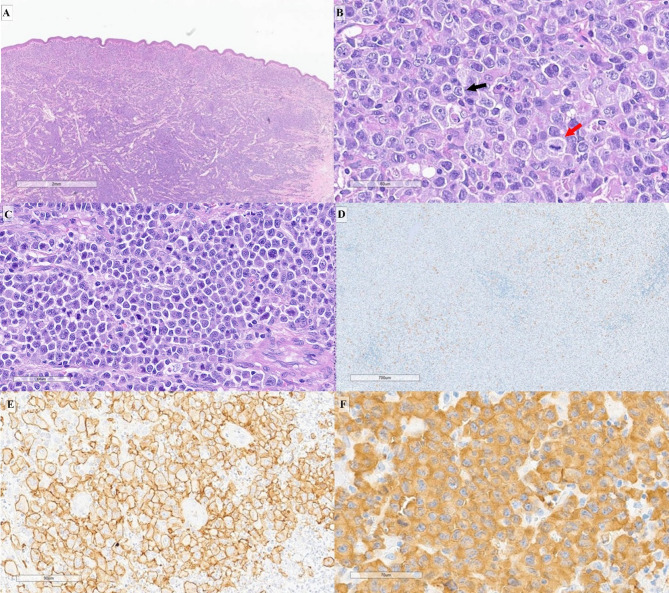
(**A**) Morphological features of primary cutaneous anaplastic large cell lymphoma, with the tumor localized in the subepidermal layer (H&E stain). (**B**) Hallmark cells displaying horseshoe-shaped nuclei (black arrow) and mitoses (red arrow) (H&E stain). (**C**) Systemic anaplastic large cell lymphoma demonstrating a diffuse pattern, predominantly of the small cell type (H&E stain). (**D**) Scattered ALK expression with an inflammatory background, characteristic of classical Hodgkin lymphoma-like anaplastic large cell lymphoma (IHC stain). (**E**) Strong CD30 positivity observed in the cell membrane (IHC stain). (**F**) Positive cytoplasmic staining for ALK (IHC stain).

### Histopathological characteristics of ALCL

The histology and IHC of ALCL cases are illustrated in Fig. 2. Most ALCL cases in this study, particularly pc-ALCL, displayed diffuse architectural patterns, while systemic ALCL showed greater variability, including some nodular patterns. As displayed in Table 2, the common morphological features of ALCL were observed in the majority of cases, with only one case exhibiting CHL-like morphology.

**Table 2:** Histopathological features of cutaneous and systemic anaplastic large cell lymphoma

	ALCL (*N* = 93)					
	Cutaneous ALCL (*n* = 13)		Systemic ALCL (*n* = 80)			
	n (%)		ALK-positive (*n* = 43)	ALK-negative (*n* = 37)	*p*-value	OR (95% CI)
			n (%)	n (%)		
**Pattern**						
Diffuse	13 (100)		37 (53.6)	32 (46.4)	0.729**	N/A
Nodular	0 (0)		4 (44.4)	5 (55.6)		
Sinusoidal	0 (0)		1 (100)	0 (0)		
CHL-like	0 (0)		1 (100)	0 (0)		
**Apoptosis**						
Present	4 (30.8)		12 (57.1)	9 (42.9)	0.717*	1.2 (0.4–3.3)
Absent	9 (69.2)		31 (52.5)	28 (47.5)		
**Necrosis**						
Present	6 (46.2)		14 (50.0)	14 (50.0)	0.622*	0.8 (0.3–2.0)
Absent	7 (53.8)		29 (55.8)	23 (44.2)		
**Morphological features**						
Common	11 (84.6)		37 (56.1)	29 (43.9)	0.301**	1.3 (0.3–5.7)
Giant cell	2 (15.4)		5 (55.6)	4 (44.4)		Ref
Small-cell	0 (0)		1 (20.0)	4 (80.0)		4.0 (0.3–5.3)
**Inflammation**						
Present	2 (15.4)		30 (61.2)	19 (38.8)	0.092*	2.2 (0.9–5.5)
Absent	11 (84.6)		13 (41.9)	18 (58.1)		
**Perinodal extension**						
Present	N/A		3 (18.7)	13 (81.3)	0.002**	0.1 (0.0–0.5)
Absent	N/A		40 (62.5)	24 (37.5)		
**Fibrotic**						
Present	7 (53.8)		22 (47.8)	24 (52.2)	0.216*	0.6 (0.2–1.4)
Absent	6 (46.2)		21 (61.8)	13 (38.2)		
**Starry-sky appearance**						
Present	2 (15.4)		8 (80.0)	2 (20.0)	0.075**	4.0 (0.8–20.2)
Absent	11 (84.6)		35 (50.0)	35 (50.0)		
**ALK staining location**						
Cytoplasm	N/A		17 (39.5)	N/A	N/A	N/A
Cytoplasm and nucleus	N/A		26 (60.5)	N/A		

Abbreviations: ALCL, Anaplastic Large Cell Lymphoma; OR, Odd Ratio; 95% CI, 95% Confidence Interval; CHL, Classic Hodgkin Lymphoma; N/A, Not Applicable

*Chi-Square; **Fisher Exact

All 93 cases in this study demonstrated CD 30 expression in more than 75% of the tumor cells. The IHC profiles are presented in Table 3. MUM1 significantly differed between the two subtypes, with MUM1 expression inversely associated with ALK negativity (*p* = 0.010, 95% CI: 1.5–37.4, OR = 7.6). The median Ki-67 expression was 80%, ranging from 40 to 95%. In pc-ALCL and ALK-negative systemic ALCL, the median Ki-67 expression was 80%, while ALK-positive cases showed a slightly lower median value of 72.5%.

**Table 3:** Immunohistochemistry pattern of all anaplastic large cell lymphoma cases

	ALCL (*N* = 93)					
	Cutaneous ALCL (*N* = 13)		Systemic ALCL (*N* = 80)			
	n (%)		ALK-positive (*n* = 43)	ALK-negative ( *n* = 37)	*p*-value	OR (95% CI)
			n (%)	n (%)		
**CD3**						
Positive	9 (69.2)		26 (52.0)	24 (48.0)	0.685*	0.8 (0.3–2.1)
Negative	4 (30.8)		17 (56.7)	13 (43.3)		
**CD43**						
Positive	8 (61.5)		38 (55.9)	30 (44.1)	0.532**	1.8 (0.5–6.1)
Negative	5 (38.5)		5 (41.7)	7 (58.3)		
**CD56**						
Positive	2 (15.4)		11 (50.0)	11 (50.0)	0.679*	0.8 (0.3–2.2)
Negative	11 (84.6)		32 (55.2)	26 (44.8)		
**CD45**						
Positive	11 (84.6)		36 (52.2)	33 (47.8)	0.533**	0.6 (0.2–2.3)
Negative	2 (15.4)		7 (63.6)	4 (36.4)		
**MUM1**						
Positive	10 (76.9)		41 (60.3)	27 (39.7)	0.010**	7.6 (1.5–37.4)
Negative	3 (23.1)		2 (16.7)	10 (83.3)		

Abbreviations: ALCL, Anaplastic Large Cell Lymphoma; OR, Odd Ratio; 95% CI, 95% Confidence Interval

*Chi-Square; ** Fisher Exact

### Prognostic assessment of ALCL patients

Among the 26 cases that were followed up, 9 survived, and 17 had deceased. Among the 9 surviving patients, 1 received CHOP, 1 underwent doxorubicin-ifosfamide, 1 had unspecified chemotherapy, and 6 lacked treatment records. Among 17 deceased patients, 8 received chemotherapy (mainly CHOP, ADOC, and methotrexate), 2 also had radiotherapy, and 9 had no documented treatment information.

Systemic ALCL had 7 surviving patients and 13 deaths, while pc-ALCL had 2 surviving patients and 4 deaths. ALK-negative patients had a life expectancy of 23 months, whereas ALK-positive patients had 28 months, and pc-ALCL patients had 11 months. B symptoms and ECOG-PS of 3–4 were significantly associated with mortality. As presented in Table 4, no statistical differences were found between IPI score, clinical stage, tumor location, and ALK expression with disease outcome. The ALK staining location also revealed no significant differences.

**Table 4:** Prognostic factors in the outcome of all anaplastic large cell lymphoma cases

	Alive (*n* = 9)	Death (*n* = 17)	*p*-value*
	*n* (%)	*n* (%)	
**B symptoms**			
Present	3 (17.6)	14 (82.4)	0.028**
Absent	6 (66.7)	3 (33.3)	
**ECOG– PS**			
0–2	9 (50.0)	9 (50.0)	0.023**
3–4	0 (0)	8 (100)	
**IPI**			
Low to low-intermediate	7 (46.7)	8 (53.3)	0.217**
Intermediate-high to high	2 (18.2)	9 (81.8)	
**Stage**			
Early	3 (23.1)	10 (76.9)	0.411**
Advanced	6 (46.2)	7 (53.8)	
**Location**			
Nodal	6 (46.2)	7 (53.8)	0.411**
Extranodal	3 (23.1)	10 (76.9)	
**ALK**			
Positive	4 (44.4)	5 (55.6)	0.667**
Negative	5 (29.4)	12 (70.6)	

Abbreviations: ALCL, Anaplastic Large Cell Lymphoma; IPI, International Prognostic Index; ECOG– PS, Eastern cooperative oncology group performance status

** Fisher Exact

## Discussion

ALCL is a rare NHL subtype, accounting for 0.06% of all malignancies, 3.3–4.4% of NHL, and 10.2–17.5% of PTCL [11–14]. This study identified 93 cases of ALCL from 2013 to 2023, comprising 13.9% pc-ALCL and 86.1% systemic ALCL, with systemic ALCL being more prevalent. Studies show systemic ALCL is more prevalent than pc-ALCL, and its incidence differs based on race, with Asians having a lower rate compared to whites [15]. This may be attributed to genetic susceptibility, lifestyle, environmental factors, and infections [16]. Notably, no cases of BIA-ALCL were observed, potentially due to the low prevalence of breast implant use in Indonesia.

pc-ALCL accounts for 14% of the ALCL cases in the present cohort, which is lower than in other Western and Asian studies [17, 18]. This may be attributable to the diagnostic challenges associated with skin lesion evaluation [19, 20]. pc-ALCL predominantly affects older adults, with a median age of 57 years, which is consistent with our findings [21, 22]. Lack of ALK translocation in pc-ALCL may exhibit rare cytoplasmic ALK positivity [23]. Morphologically, pc-ALCL resembles systemic ALK-negative ALCL, with over 75% of tumor cells expressing strong CD30 positivity [24]. 

The distinction between ALK-positive and ALK-negative systemic ALCL stems from a t(2;5) translocation involving *ALK* and *NPM* genes, leading to oncogenic activation [25–27]. Our cohort reported systemic ALK-positive and ALK-negative rates of 53.7% and 46.3%, consistent with global studies, with a slightly higher rate observed in Asian populations [11, 13, 21]. ALK-positive cases, more frequent in younger patients, showed dual cytoplasmic and nuclear staining in 60.5% of cases, linked to better five-year overall survival (OS) [5, 28–31]. Consistent with other studies, ALK-negative cases occurred in older individuals, with greater heterogeneity and poorer prognosis [32, 33]. Despite ALK-positive patients having a slightly longer life expectancy (28 vs. 23 months), overall outcomes between subtypes showed no significant difference.

Systemic ALCL presented with significant Ann-Arbor staging and nodal involvement, particularly in ALK-positive cases (40% in the head and neck), which is consistent with previous studies [34, 35]. This study found extranodal involvement, including ocular sites, with certain studies exploring this phenomenon [36, 37]. Systemic ALCL with ALK-positive expression present early and have better survival rates, aligning with these research findings [38]. The oncogenic properties of the ALK fusion protein may link ALK expression to B symptoms in ALCL [5, 39–41]. Prior studies reported lower B symptoms in ALK-negative cases; our cohort showed higher, though insignificant, statistics [13, 21]. ALK-negative patients had higher IPI scores, poorer OS, and elevated ECOG-PS, which was the only statistically significant prognostic factor in our study, differing from previous findings [42, 43]. 

Our study confirmed ALCL unique histopathological features, with diffuse patterns predominating, consistent with previous studies [12]. Unusual CHL-like patterns and rare small-cell variants were found in this research, which complicates diagnosis [44, 45]. Giant cell morphology was less frequent (9 cases) compared to King et al. study of 30% [46]. CD30 positivity remains a key marker, while CD3 and CD45 showed higher prevalence in ALK-positive cases than in earlier reports [47]. 

This study identifies a significant link between negative MUM1 expression and ALK-negative systemic ALCL, unlike previous reports [48–50]. While MUM1 (known as interferon regulatory factor-4/IRF4) aids lymphoma subtyping, its role in systemic ALCL remains unclear [51]. This study is the first to show a big difference in MUM1 IHC expression between ALK-positive and ALK-negative cases. The discrepancies in outcomes may be attributable to varying sample sizes; further studies are necessary.

Chemotherapy, particularly CHOP (cyclophosphamide, doxorubicin, vincristine, prednisone), remains the standard treatment for ALCL. In this cohort, 14 patients received CHOP, with two also undergoing radiotherapy. A study reported better 3-year progression-free survival (PFS) and OS rates in ALK-positive cases (66% and 82%) compared to ALK-negative cases (35% and 44%), despite 40–65% relapse rates [52, 53]. The addition of etoposide (CHOEP) improved outcomes, and brentuximab vedotin showed an 86% response in relapsed cases but is rarely used in Indonesia due to high costs [54–58]. 

This study highlights the clinicopathological features of ALCL diagnosed over a decade at a tertiary public general hospital affiliated with the biggest medical teaching institution in Indonesia. The results emphasize the predominance of ALK-positive systemic ALCL and its favorable outcomes. These findings highlight the importance of standardized treatment protocols and comprehensive multicenter data collection in managing rare lymphomas such as ALCL.

### Limitations

Despite providing valuable real-world insights into ALCL, the lack of comprehensive clinical and therapeutic information and a small survival cohort limit the interpretability of the conclusions, particularly prognostic factors and survival outcomes. The single-center setting at a national referral hospital may lead to referral bias, potentially limiting the generalizability of the results.

## Electronic supplementary material

Below is the link to the electronic supplementary material.

Supplementary Material 1

## Data Availability

The datasets used and/or analysed during the current study are available from the corresponding author on reasonable request.

## Abbreviations

ADOC: Doxorubicin (Adriamycin), cisplatin (cis-Diamminedichloroplatinum), vincristine (Oncovin), cyclophosphamide
ALCL: Anaplastic Large Cell Lymphoma
ALK: Anaplastic Lymphoma Kinase
BIA-ALCL: Breast-Implant-Associated ALCL
CHL-like: Classical Hodgkin Lymphoma-Like
CHOEP: Cyclophosphamide, Doxorubicin, Vincristine, Etoposide, and Prednisone
CHOP: Cyclophosphamide, doxorubicin, vincristine, and prednisone
ECOG-PS: Eastern Cooperative Oncology Group Performance Status
H&E: Hematoxylin and Eosin
IHC: Immunohistochemical
IPI: International Prognostic Index
IRF4: Interferon Regulatory Factor
LDH: Lactate Dehydrogenase
MUM1: Multiple Myeloma 1
NHL: Non-Hodgkin Lymphoma
OS: Overall Survival
pc-ALCL: Primary Cutaneous ALCL
PFS: Progression-Free Survival
SPSS: Statistical Package for the Social Sciences
UI-CMH: Universitas Indonesia-Dr. Cipto Mangunkusumo Hospital
WHO: World Health Organization
