# Supplementary material for: Comprehensive study of anaplastic large cell lymphoma: clinicopathological features from Indonesia
Source: BMC Res Notes. 2025 Jul 9;18:282. doi: 10.1186/s13104-025-07354-5 (PMC12243422; doi:10.1186/s13104-025-07354-5)
Supplement: Supplementary file 1 — Supplementary Material 1 [file 13104_2025_7354_MOESM1_ESM.docx]

Supplement tables :

| **Antibody** | **Clone** | **Provider** | **Dilution** |
| --- | --- | --- | --- |
| AE1/3 | AE1/AE3 | Leica | RTU |
| ALK-1 | 5A4 | BIOCARE | 1 : 100 |
| BCL2 | 124 | Cell Marque | 1 : 100 |
| BCL6 | LN22 | BIOCARE | 1 : 100 |
| BOB-1 | TG14 | BIOCARE | 1 : 50 |
| CD10 | 56C6 | Leica | 1 : 50 |
| CD123 IL-3Ra | BSB-59 | BioSB | 1 : 50 |
| CD138 | EP201 | Cell Marque | 1 : 150 |
| CD15 | BSB-119 | BioSB | 1 : 200 |
| CD16 | 2H7 | Leica | 1 : 200 |
| CD163 | 10D6 | BIOCARE | 1 : 200 |
| CD1a | MTB1 | Leica | 1 : 50 |
| CD20 | L26 | SCYTEK | 1 : 100 |
| CD21 | 2G9 | Cell Marque | 1 : 200 |
| CD23 | 1B12 | Leica | 1 : 50 |
| CD3 | PS1 | BIOCARE | 1 : 100 |
| CD30 | Ber-H2 | Cell Marque | 1 : 50 |
| CD31 | JC70 | Cell Marque | 1 : 50 |
| CD33 | PWS44 | BIOCARE | 1 : 50 |
| CD34 | QBEnd/10 | BioSB | 1 : 75 |
| CD38 | SPC32 | Leica | 1 : 75 |
| CD4 | 4B12 | BIOCARE | 1 : 200 |
| CD43 | MT1 | Cell Marque | 1 : 50 |
| CD45ro | UCHL-1 | BIOCARE | RTU |
| CD5 | RBT-CD5 | BioSB | 1 : 200 |
| CD56 | 123C3.D5 | BioSB | 1 : 100 |
| CD61 | 2f2 | Cell Marque | 1 : 100 |
| CD64 | OTI3D3 | ABCAM | 1 : 50 |
| CD68 | Kp-1 | Cell Marque | 1 : 150 |
| CD7 | LP15 | BIOCARE | 1 : 400 |
| CD71 | H68.4 | BIOCARE | RTU |
| CD79a | JCB117 | DAKO | 1 : 300 |
| CD8 | SP16 | BIOCARE | 1 : 100 |
| CD99 | PCB1 | Leica | 1 : 200 |
| C-MYC | EP121 | Cell Marque | 1 : 50 |
| CYCLIN D1 | SP4 | BIOCARE | 1 : 100 |
| GATA3 | L50-823 | Cell Marque | 1 : 500 |
| GRANZYME B | GrB | BioSB | 1 : 30 |
| HHV-8 | 13B10 | Cell Marque | 1 : 200 |
| HMB45 | HMB-45 | BioSB | 1 : 50 |
| ICOS/CD278 | RM417 | BioSB | 1 : 100 |
| IgD | EP173 | Cell Marque | 1 : 50 |
| IgG | Polyclonal | DBS | 1 : 200 |
| IgG4 | HP6025 | BIOCARE | 1 : 100 |
| IgM | R1/69 | DBS | 1 : 100 |
| KAPPA | L1C1 | BIOCARE | 1 : 500 |
| KI-67 | SP6 | DBS | 1 : 50 |
| LAMBDA | EP172 | Cell Marque | 1 : 500 |
| LCA (CD45) | PD7/26 & 2B11 | SCYTEK | 1 : 100 |
| LEF-1 | EP310 | BioSB | 1 : 100 |
| MPO | Polyclonal | BIOCARE | 1 : 1.000 |
| MUM-1 | EAU32 | LEICA | 1 : 200 |
| MyD88 | polyclonal | BOSTER | RTU |
| PAX-5 | BC/24 | BIOCARE | 1 : 100 |
| PAX-8 | ZR-1 | BioSB | 1 : 100 |
| PDX1 | EP139 | BioSB | 1 : 100 |
| PERFORIN | MRQ-23 | Cell Marque | 1 : 50 |
| SOX-11 | CLO142 | BioSB | 1 : 150 |
| T-bet / Tbx21 | 4B10 | ABCAM | 1 : 200 |
| TdT | SEN28 | LEICA | 1 : 100 |
| TIA-1 | TIA-1 | BIOCARE | 1 : 200 |
| LMO2 | SP51 | Biocare | RTU |
| CXCL13 | POLYCLONAL | VITRO | RTU |
